# How Do Qataris Source Health Information?

**DOI:** 10.1371/journal.pone.0166250

**Published:** 2016-11-10

**Authors:** Sopna M. Choudhury, Teresa Arora, Seham Alebbi, Lina Ahmed, Abdi Aden, Omar Omar, Shahrad Taheri

**Affiliations:** 1 Department of Medicine and Clinical Research Core, Weill Cornell Medicine in Qatar, Qatar Foundation-Education City, Doha, Qatar; 2 Department of Medicine, Hamad Medical Corporation, Doha, Qatar; Brighton & Sussex Medical School, Falmer, UNITED KINGDOM

## Abstract

**Background:**

Qatar is experiencing rapid population expansion with increasing demands on healthcare services for both acute and chronic conditions. Sourcing accurate information about health conditions is crucial, yet the methods used for sourcing health information in Qatar are currently unknown. Gaining a better understanding of the sources the Qatari population use to recognize and manage health and/or disease will help to develop strategies to educate individuals about existing and emerging health problems.

**Objective:**

To investigate the methods used by the Qatari population to source health information. We hypothesized that the Internet would be a key service used to access health information by the Qatari population.

**Methods:**

A researcher-led questionnaire was used to collect information from Qatari adults, aged 18–85 years. Participants were approached in shopping centers and public places in Doha, the capital city of Qatar. The questionnaire was used to ascertain information concerning demographics, health status, and utilization of health care services during the past year as well as sources of health information used.

**Results:**

Data from a total of 394 eligible participants were included. The Internet was widely used for seeking health information among the Qatari population (71.1%). A greater proportion of Qatari females (78.7%) reported searching for health-related information using the Internet compared to Qatari males (60.8%). Other commonly used sources were family and friends (37.8%) and Primary Health Care Centers (31.2%). Google was the most commonly used search engine (94.8%). Gender, age and education levels were all significant predictors of Internet use for heath information (P<0.001 for all predictors). Females were 2.9 times more likely than males (P<0.001) and people educated to university or college level were 3.03 times more likely (P<0.001) to use the Internet for heath information.

**Conclusions:**

The Internet is a widely used source to obtain health-related information by the Qatari population. Internet search engines can be utilized to guide users to websites, developed and monitored by healthcare providers, to help convey reliable and accurate health information to Qatar’s growing population.

## Introduction

A challenge for modern healthcare services is the provision of easily accessible, accurate, and timely information for patients and the public. Chronic disorders, such as obesity[1] and type 2 diabetes mellitus[2], are of particular concern, because of their increasing prevalence worldwide. To prevent, delay, and manage these serious and costly chronic disorders, there is a need for continual public and patient education. This requires patients and the public to be directed to relevant and accurate information. For acute medical conditions, accurate information can help effective and timely action for identification of serious conditions, while for less serious conditions, it may prevent unnecessary usage of limited healthcare resources.

Whilst chronic conditions place a major burden on healthcare systems and impede quality of life in the affected individuals, acute illnesses are also of interest. For example, people are likely to independently search for health-related information, and self-medicate for acute illnesses rather than taking the time to visit a health professional [3]. Sourcing accurate information about health conditions, acute or chronic, is crucial as this may help establish healthier behaviors amongst the population [4]. The provision of information on health has changed dramatically with greater reliance on sources outside the traditional healthcare services where healthcare professionals are the sole authority on disease management[5]. With increasing prevalence of chronic disorders, interaction between patients and healthcare professionals is limited and greater reliance is placed on self-management. Increasing reliance is placed on information sources such as the Internet.

Like many countries in the Middle East and North Africa region, Qatar has experienced rapid development and population expansion. Chronic disorders have become highly prevalent in Qatar, while greater demands are placed on acute medical services. There is currently no information regarding how the population in Qatar seeks information regarding health issues. In particular, the role of the Internet for sourcing health information by the Qatari population is unknown. Technology is widely accessible and utilized in Qatar [6], and Internet use has increased from 41% of households in 2008 to 70% in 2010 [7]. The purpose of our study was to investigate the methods used to source health information, in any language, by the Qatari population. We hypothesized that the Internet would be the main provider of health information for Qatari adults. Identifying methods for sourcing health information by the Qatari population will be a key step towards directing the public and patients to the necessary information to tackle the country’s growing healthcare challenges.

## Methods

This was a survey based cross-sectional study and recruited adults only, with no identifiable data collection. Ethical exemption for the survey was granted from Hamad Medical Corporation/Weill Cornell Medical College in Qatar Joint Institutional Review Board (IRB) (14–00017). Written informed consent was not required, as determined by the IRB. Consent to participate was obtained verbally and noted by completion of questionnaire. Male and female Qatari adults were approached in shopping centers, education establishments, and public places in Doha, Qatar. Male researchers approached male Qatari participants where possible and female researchers only approached female Qatari participants to comply with cultural norms. Those who met the inclusion criteria (males or females; aged 18–85 years old; Qatari National; Arabic speaking) based on visual inspection, and provided verbal consent, completed a researcher-led questionnaire. The questions in the survey were from combined previous research about Internet use for health and modified in order to fit with the culture in Qatar. The questionnaire was used to ascertain basic information concerning: demographics, health status, utilization of healthcare services during the past year and resources utilized to acquire health/disease information.

### Statistical analysis

All statistical analyses were performed using Stata version 13.0. We compared potential differences between gender and age, education, self-reported health status and the number of Primary Health Care Centre (PHCC) visits in the previous 12 months. We then investigated possible differences between gender and resources of health information. We assessed the most common search engine used as a means of seeking health information amongst Internet users. Chi-squared tests were performed for unordered categorical variables and Chi-squared tests for trend were used for ordinal categorical variables. Logistic regression was conducted using gender, age, education level and health status as predictor variables to determine which of these factors influence the use of the Internet for seeking health information. A P value of <0.05 was considered to be statistically significant.

## Results

A total of 403 questionnaires were completed. Three-hundred and ninety-four eligible questionnaires were included in the analysis. The demographics of the study participants are provided in Table 1. The majority (67.9%) of the participants were aged between 18–34 years. More men rated their health as very good compared to women (60.1% and 53.1%, respectively), although this difference did not reach statistical significance χ^2^(3, 387) = 5.7, p = 0.319.

**Table 1:** Study sample characteristics of 394 Adult Qatari participants, according to gender.

	Men (n = 148)	Women (n = 241)	Total (n = 394)*
*Age*, *n (%)*			
18–34 years	98 (66.2)	166 (69.2)	267 (67.9)
35–59 years	49 (33.1)	68 (28.3)	118 (30.1)
60+ years	1 (0.7)	6 (2.5)	8 (2.0)
Missing	0	1	1
*Education (highest level completed)*, *n (%)*			
Up to secondary school	59 (41.0)	99 (41.6)	159 (41.2)
College/University	84 (58.3)	138 (58.0)	225 (58.3)
None	1 (0.7)	1 (0.4)	2 (0.5)
Missing	4	3	8
*Self-reported Health Status*, *n (%)*			
Very good	89 (60.1)	127 (53.1)	216 (55.1)
Good	48 (32.4)	101 (42.3)	153 (39.0)
Poor	8 (5.4)	10 (4.2)	19 (4.9)
Very poor	3 (2.0)	1 (0.4)	4 (1.0)
Missing	0	2	2
*Number of visits to PHCC in last 12 months*, *n (%)*			
0	20 (13.6)	35 (14.6)	55 (14.0)
1–3	74 (50.3)	122 (50.8)	198 (50.5)
4–5	33 (22.5)	46 (19.2)	82 (20.9)
6+	20 (13.6)	37 (15.4)	57 (14.5)
Missing	1	1	2
*Source of seeking information on health concerns*, *n (%)*			
Internet	90 (60.8)	190 (78.7)	280 (71.1)
Family/friends	60 (40.5)	87 (36.1)	149 (37.8)
TV/radio	15 (10.1)	19 (7.9)	34 (8.6)
PHCC	48 (32.4)	73 (30.3)	123 (31.2)
Books	14 (9.5)	23 (9.5)	37 (9.4)
Magazines	8 (5.4)	13 (5.4)	22 (5.6)
Newspapers	15 (10.1)	8 (3.2)	23 (5.8)
Mobile Application	14 (9.5)	27 (11.2)	41 (10.4)
*Reasons for not using Internet*, *n (%)*			
No access	1 (1.8)	8 (15.1)	9 (8.0)
Unreliable/inaccurate	31 (56.4)	33 (62.2)	65 (57.5)
Cannot use technology	3 (5.5)	2 (3.8)	5 (4.4)
Lack of time	13 (23.6)	8 (15.1)	22 (19.5)
Impersonal	4 (7.3)	2 (3.8)	6 (5.3)
Lack of information	4 (7.3)	1 (1.9)	7 (6.2)
Language barrier	3 (5.6)	2 (3.8)	6 (5.3)
Other	3 (5.6)	4 (7.9)	7 (6.3)
*Search engine used*, *n (%)*			
Google	72 (93.5)	145 (95.4)	217 (94.8)
Other	5 (6.5)	7 (4.6)	12 (5.2)
Missing	71	89	165

* 5 participants had missing gender information;

PHCC = Primary Health Care Center.

The sources of health information used by Qatari adults, according to gender, are shown in Table 2. Eight percent of the surveyed population did not have access to the Internet. A large proportion of the participants (71.1%) used the Internet to seek health-related information. A significantly higher proportion of women (78.7%) compared to men (60.8%) used the Internet to source health-related information, χ^2^(1, 72) = 14.8, P<0.001. Other commonly used sources were family and friends (37.8%) and PHCCs (31.2%).

**Table 2:** Sources of health information used by Qatari Adults, according to gender.

Sources of information	Men (n = 148)	Women (n = 241)	Total (n = 389)*	P value
Internet, n (%)	90 (60.8)	190 (78.7)	280 (71.1)	<0.0001
Family/friends, n (%)	60 (40.5)	87 (36.1)	149 (37.8)	0.380
TV/Radio, n (%)	15 (10.1)	19 (7.9)	34 (8.6)	0.445
PHCC, n (%)	48 (32.4)	73 (30.3)	123 (31.2)	0.658
Books/Magazines/Newspapers, n (%)	30 (20.3)	31 (12.9)	61 (15.7)	0.051

* 5 participants had missing gender information;

PHCC = Primary Health Care Center.

Of all the participants who used the Internet to source health information, the majority (94.8%) used Google as the main source of searching. The reasons for non-Internet use included the belief that that the information was inaccurate (30.1%) or unreliable (27.4%). Only 4.4% of the study population said they were not able to use technology (Table 2).

Logistic regression was performed to assess the impact of a number of factors on online health information seeking behavior. Table 3 presents the results from multivariate analysis. Health status was not found to be an indicator of online health information usage. However, gender, age and education levels were found to be indicators of online health information usage, in both univariate and multivariate analyses. Women were 2.9 times more likely to use the Internet than men.

**Table 3:** Indicators of online health information usage.

Factor	OR (95% CI)	P value*
*Gender*		<0.001
Male	1.00 (reference)	
Female	2.90 (1.75–4.81)	
*Age (years)*		<0.001
18–24	1.00 (reference)	
25–34	0.74 (0.39–1.40)	
35–44	0.54 (0.26–1.10)	
45–59	0.17 (0.08–0.39)	
60+	0.18 (0.03–1.13)	
*Education*		<0.001
Secondary	1.00 (reference)	
College or University	3.03 (1.80–5.10)	
*Health Status*		0.430
Poor/very poor	1.00 (reference)	
Good/very good	0.62 (0.19–2.03)	

OR = Odds Ratio; CI = Confidence Interval.

* P value obtained from logistic regression analysis.

## Discussion

Our study aimed to explore the methods used to source health information, in any language, by the Qatari population. We found that the Internet, Primary Health Care Centers (PHCC) and family/friends are the most common sources of health information used by the Qatari population. We also observed that more women used the Internet than men, and the level of education, and age were predictors of sourcing health information on the Internet.

We found that the Internet is a widely used source for seeking health information amongst the surveyed Qatari population (71.1%), above and beyond PHCCs. This result is similar to rates found in the US, where Kontos and colleagues found 79.04% of online Americans reported using the Internet to seek health information for themselves and 57.04% had used it for someone else [8].

A significantly higher proportion of Qatari females reported using the Internet to seek health-related information compared to Qatari males. This result is in agreement with previous studies [9–13]. Atkinson and colleagues, using multivariate analysis, reported that women were more likely to seek health information online (OR = 2.23, 95% CI: 1.60–3.09) compared to men[14]. Weaver and colleagues found in their study on health information–seeking behaviors, that more women (60.5%) than men (39.5%) obtained health information from the Internet (OR = 1.99; 95% CI = 1.42, 2.79)[15]. The above findings are echoed in the usage of traditional healthcare services. Wang and colleagues observed similar findings in consultation rates in primary care, where men were 32% less likely than women to visit primary health care centers for a consultation[16]. Women have generally been more likely to obtain health information than men, whether online or in face to face consultations. This may be because women assume the role of childrearing in the majority of cases and also spend more time caring for sick spouses and children[11]. Women may also monitor and respond to their bodily functions more than men[11], prompting them to consult health care practitioners. Another reason for consultations is that younger women require more health engagement/information due to issues related to reproduction[16]. Also, men tend to be more unaware of health information seeking methods due to social constructions of gender in terms of perceived roles and masculinity, as identified by Addis and Mahalik[17]. Previous research has shown that masculinity traits deter from help seeking behaviors such as asking questions at appointments[18].

Our study noted that family and friends play a major role as a source of health information (37.8%). However, this figure seems to be less than that found in the US, where 56% and 65% of adults, reporting having no chronic conditions and one or more chronic conditions respectively, obtained information or support from family and friends[19].

Primary care services were utilized by 31.2% of Qatari participants as a source of health-related information. A previous US study found that primary care physicians and nurses were reported to be the most useful source of health information[20]. The preference of seeking medical advice from health professionals may be reflective of an individual’s belief that fits in with the traditional notions where the health care professional is respected and trusted[5,21,22]. The primary care utilization in our study also complements the Qatar National Health Strategy (QNHS) 2011–2016 [23] to fulfill the Qatar National Vision 2030[24]. The aim of the QNHS is to make primary care the cornerstone of a person-centered health system in Qatar, in line with the World Health Organization (WHO), which recognizes that health systems oriented towards primary health care are more likely to cost effectively deliver better health outcomes and greater public satisfaction[25].

Our study found that Google was the most commonly used Internet search engine (55.8%) to obtain health-related information as found in previous work from Saudi Arabia on patients with type 2 diabetes patients, where Google was used by 98% of the studied population[26]. Some participants in our study reflected their aversion to using the Internet as a source of collecting health-related information. They attributed this to many pre-defined reasons provided on the questionnaire, which included: inaccuracy; time constrains; unreliability; subjectivity; technological challenge; language barrier, information insufficiency, and inaccessibility.

The level of education was found to be a significant predictor of Internet health information usage in our sample. People with college or university level education were 3.03 times more likely to use the Internet for health information than people with lower education levels. This reinforces previous findings by AlGhamdi and Moussa in their study on Internet use to search for health related information by the public[12]. They observed that people with university or higher level of education used the Internet more than those with high school or lower education (OR = 1.7, 95% CI 1.1–2.8). Kontos and colleagues also observed that those with high school degree or less and those with some college education levels were 35% less likely to have used the Internet than that college graduates (OR 0.64, 95% CI 0.42–0.98 and OR 0.67, 95% CI 0.49–0.93, respectively)[8].

We also observed that age was a predictor of sourcing health information on the Internet, with the younger adults using the Internet significantly more, similar to previous findings[12,27]. This may be due to the availability and rise in technology use, where younger people are more familiar with technology as they have grown up with technology. Also, older people may be more apprehensive about technology use[8,28].

Self-reported poorer health status was not associated significantly with more online use. This finding does not agree with previous findings where overall health condition has been linked to the illness behaviour model[29], indicative of poor health individuals being more likely to seek health information on the Internet. The reason for our findings could be the participation of a younger more healthy population in the study.

### Limitations

There are a number of limitations to our study. The majority (86.5%) of our sample was less than 45 years old. Whilst our study is the first to investigate methods used for sourcing health-related information in Qatar, and benefits from a relatively large sample size, future studies could obtain similar data from a larger sample and include older individuals. The younger age in our sample, however, is a reflection of the Qatari population. Collecting data on medical history may help to explain the sources used to obtain health information, as chronic disease management or a recent diagnosis of an illness, such as cancer, have been found to be strong motivators for Internet use. Also, it would be interesting to examine whether the population uses different sources of health-information depending on the type of information they are seeking, for example using the Internet to search for sensitive data, as found in adolescents[30]. We did not capture whether the information searched for was for the participant themselves or for family and friends. Our study relied on self-report, which may be subject to a number of biases (recall, social desirability). Furthermore, our study was cross-sectional. A prospective study is required to determine if individuals consistently use the same tools for identifying health information or not and the reasons behind this, which could be qualitatively explored. Finally, the type and amount of information, as well as how this information is sourced, is affected by personal as well as situational factors. It would be useful to collect more data on socio-economic, educational background, health literacy and demographics to see whether these have any links to the sources that are used for health-information seeking as previous studies have shown that these characteristics may influence behavior[31]. Also, it would be useful to collect information on the language the health information is searched for on the Internet as Jamal and colleagues found a majority of their participants searched in Arabic only[26].

## Conclusion

We present findings from the first study to explore the methods used for sourcing health information by the Qatari adult population. Although many studies have examined the use of Internet for health information, our study is the first in Qatar to examine this. We have demonstrated that the Internet was the most widely used source amongst the Qatari population. Significantly more Qatari women use the Internet to search for health-related information compared to Qatari men. Google was the most commonly used search engine.

In the future, we anticipate that the results from this study can be used to create a means to guide the Qatari population towards obtaining the most accurate information related to their health using reliable Internet sources. Also, the Internet can be used as a source of health promotion, to help the country achieve its Qatar National Vision 2030, and to establish healthier behaviors amongst its population. Health professionals and organizations could use the growing practice of Internet health information seeking behavior to their advantage, by preparing their own websites to direct patients to reliable, accurate and culturally appropriate information in Arabic [31] and English. This could potentially help with shared decision-making and help improve treatment adherence, patient satisfaction, quality of life and well-being. Our findings are also in line with existing research across different parts of the world, where there has been an increase in technology use, leading to increased health sourcing activity on the Internet. However, it is important to remember that this may not be a suitable method of sourcing heath information for all healthcare users and a multi-component method of accurate and reliable information is required in order to engage with all groups of people.

## Supporting Information

S1 File This is the questionnaire used for the data collection. (DOCX)

## References

[1] Ng M, Fleming T, Robinson M, Thomson B, Graetz N, Margono C, et al Global, regional, and national prevalence of overweight and obesity in children and adults during 1980–2013: a systematic analysis for the Global Burden of Disease Study 2013. The Lancet. 2014 8;384(9945):766–81. 10.1016/S0140-6736(14)60460-8 PMC4624264 24880830

[2] International Diabetes Federation. IDF Diabetes atlas- Sixth edition [Internet]. International Diabetes Federation; 2013. Available http://www.idf.org/sites/default/files/EN_6E_Atlas_Full_0.pdf

[3] Gidwani R, Zulman D. Association Between Acute Medical Exacerbations and Consuming or Producing Web-Based Health Information: Analysis From Pew Survey Data. J Med Internet Res. 2015 6 23;17(6):e145 10.2196/jmir.3801 26104000 PMC4526957

[4] Dutta-Bergman MJ. Health attitudes, health cognitions, and health behaviors among Internet health information seekers: population-based survey. J Med Internet Res. 2004 5 28;6(2):e15 10.2196/jmir.6.2.e15 15249264 PMC1550593

[5] Stoeckle J, editor. Encounters between patients and doctors: An anthology. Cambridge, MA: MIT Press; 1987.

[6] Andrés L, Cuberes D, Diouf M, Serebrisky T. The diffusion of the Internet: A cross-country analysis. Telecommun Policy. 2010 6;34(5–6):323–40.

[7] Supreme Council of Information and Communication (ictQATAR). Qatar’s National ICT Plan 2015: Advancing the Digital Agenda [Internet]. Qatar: Supreme Council of Information and Communication (ictQATAR); Available http://www.scribd.com/doc/58217923/Qatar%E2%80%99s-National-ICT-Plan-2015-Advancing-the-Digital-Agenda

[8] Kontos E, Blake KD, Chou W-YS, Prestin A. Predictors of eHealth usage: insights on the digital divide from the Health Information National Trends Survey 2012. J Med Internet Res. 2014;16(7):e172 10.2196/jmir.3117 25048379 PMC4129114

[9] Powell J, Inglis N, Ronnie J, Large S. The characteristics and motivations of online health information seekers: cross-sectional survey and qualitative interview study. J Med Internet Res. 2011;13(1):e20 10.2196/jmir.1600 21345783 PMC3221342

[10] Bianco A, Zucco R, Nobile CGA, Pileggi C, Pavia M. Parents Seeking Health-Related Information on the Internet: Cross-Sectional Study. J Med Internet Res. 2013 9 18;15(9):e204 10.2196/jmir.2752 24047937 PMC3785974

[11] Manierre MJ. Gaps in knowledge: Tracking and explaining gender differences in health information seeking. Soc Sci Med. 2015 3;128:151–8. 10.1016/j.socscimed.2015.01.028 25618604

[12] AlGhamdi KM, Moussa NA. Internet use by the public to search for health-related information. Int J Med Inf. 2012 6;81(6):363–73. 10.1016/j.ijmedinf.2011.12.004 22217800

[13] Gallagher S, Doherty D. Searching for health information online: characteristics of online health seekers. J Evid-Based Med. 2009 5;2(2):99–106. 10.1111/j.1756-5391.2009.01021.x 21348996

[14] Atkinson NL, Saperstein SL, Pleis J. Using the internet for health-related activities: findings from a national probability sample. J Med Internet Res. 2009;11(1):e4 10.2196/jmir.1035 19275980 PMC2762768

[15] Weaver JB, Mays D, Weaver SS, Hopkins GL, Eroğlu D, Bernhardt JM. Health Information–Seeking Behaviors, Health Indicators, and Health Risks. Am J Public Health. 2010 8;100(8):1520–5. 10.2105/AJPH.2009.180521 20558794 PMC2901281

[16] Wang Y, Hunt K, Nazareth I, Freemantle N, Petersen I. Do men consult less than women? An analysis of routinely collected UK general practice data. BMJ Open. 2013 8 19;3(8):e003320–e003320. 10.1136/bmjopen-2013-003320 23959757 PMC3753483

[17] Addis ME, Mahalik JR. Men, masculinity, and the contexts of help seeking. Am Psychol. 2003 1;58(1):5–14. 12674814 10.1037/0003-066x.58.1.5

[18] Galdas P. Men, masculinity, and help-seeking behaviour In: Men’s Health: Body, Identity, and Social Context. Sussex, UK: Wiley-Blackwell; 2009 p. 63–82.

[19] Pew Research Centre. Part Two: Sources of Health Information | Pew Research Center [Internet]. [cited 2016 Apr 27]. Available http://www.pewinternet.org/2013/11/26/part-two-sources-of-health-information/

[20] Diaz JA, Griffith RA, Ng JJ, Reinert SE, Friedmann PD, Moulton AW. Patients’ use of the internet for medical information. J Gen Intern Med. 2002 3;17(3):180–5. 10.1046/j.1525-1497.2002.10603.x 11929503 PMC1495021

[21] Bellin LE. The shrinking autonomy of the practicing physician and its impact on quality. J Community Health. 1986;11:155–64. 3540034 10.1007/BF01338796

[22] Brown JB, Carroll J, Boon H, Marmoreo J. Women’s decision-making about their health care: views over the life cycle. Patient Educ Couns. 2002 12;48(3):225–31. 12477607 10.1016/s0738-3991(02)00175-1

[23] Supreme Council of Health, Qatar. National Health Strategy 2011–2016 [Internet]. Supreme Council of Health, Qatar; Available http://www.nhsq.info/about-the-strategy/qatar-national-vision

[24] QNV 2030 Document [Internet]. [cited 2016 May 2]. Available http://www.mdps.gov.qa/portal/page/portal/gsdp_en/qatar_national_vision/qnv_2030_document

[25] World Health Organization W. Research for universal health coverage: World health report 2013. Geneva, Switzerland: World Health Organization; 2013.

[26] Jamal A, Khan SA, AlHumud A, Al-Duhyyim A, Alrashed M, Bin Shabr F, et al Association of Online Health Information–Seeking Behavior and Self-Care Activities Among Type 2 Diabetic Patients in Saudi Arabia. J Med Internet Res. 2015 8 12;17(8):e196 10.2196/jmir.4312 26268425 PMC4642387

[27] Li N, Orrange S, Kravitz RL, Bell RA. Reasons for and predictors of patients’ online health information seeking following a medical appointment. Fam Pract. 2014 10;31(5):550–6. 10.1093/fampra/cmu034 24963151

[28] Hargittai E. Digital Na(t)ives? Variation in Internet Skills and Uses among Members of the “Net Generation.” Sociol Inq. 2010 2;80(1):92–113.

[29] Li J, Theng Y-L, Foo S. Predictors of online health information seeking behavior: Changes between 2002 and 2012. Health Informatics J. 2015 8 10; 10.1177/1460458215595851 26261218

[30] Gray NJ, Klein JD, Noyce PR, Sesselberg TS, Cantrill JA. Health information-seeking behaviour in adolescence: the place of the internet. Soc Sci Med 1982. 2005 4;60(7):1467–78. 10.1016/j.socscimed.2004.08.010 15652680

[31] Lambert SD, Loiselle CG. Health Information Seeking Behavior. Qual Health Res. 2007 10 1;17(8):1006–19. 10.1177/1049732307305199 17928475

