# Supplementary material for: How Do Qataris Source Health Information?
Source: PLoS One. 2016 Nov 10;11(11):e0166250. doi: 10.1371/journal.pone.0166250 (PMC5104325; doi:10.1371/journal.pone.0166250)
Supplement: S1 File — (DOCX) [file pone.0166250.s001.docx]

**Questionnaire**

**Part 1**

1. Are you

☐ Male

☐ Female

1. What is your age

☐ 18-24 years

☐ 25-34 years

☐ 35-44 years

☐ 45-59 years

☐ 60+ years

1. What is your nationality?

____________________________________

1. What is the highest level of education that you completed?

☐ Secondary

☐ College

☐ University

1. How would you rate your health?

☐ Very good

☐ Good

☐ Poor

☐ Very poor

1. How many visits have you made to your Primary Care Physician in the last 12 months that were related to your own health?

☐ 0

☐ 1-3

☐ 4-5

☐ 6+

1. What was the purpose of your visit to your PCP?

☐ Symptoms/pain

☐ Medication

☐ Follow up

☐ Emergency

☐ Other ________________________________________

1. How well did you understand the feedback provided to you by your PCP?

☐ Excellent

☐ Good

☐ Average

☐ (Below average)

1. What sources do you use to seek information about health concerns?

☐ Internet

☐ Family/friends

☐ TV/radio

☐ PCP

☐ Books

☐ Magazines

☐ Newspapers

☐ Mobile app

**Part 2 (if did not check internet in question 9)**

1. What are the reasons that you do not use the internet to source information about health concerns?

☐ No access

☐ Unreliable

☐ Inaccurate

☐ Can’t use tech

☐ Lack of time

☐ Impersonal

☐ Lack of info

☐ Language barrier

☐ Other _____________

1. If there was a website available that addressed your concerns above, would you be willing to use it?

☐ Yes

☐ No

☐ Don’t know

3. If not, why?

_______________________________________________________________________________________________________________________________________________________________________________________________________________

4. Would you be willing to help us with our research involving a confidential group discussion about health concerns and how to access information?

☐ Yes

☐ No

If yes, please provide details so that we can contact you

Email____________________________________________________

Mobile_______________________

**Part 2 (if they did check internet in question 9)**

1. Do you use a specific search engine or visit a specific website to find out about health concerns when using the internet?

Engine _______________________________________-____

Website ___________________________________________

1. Do you use the internet to confirm the health information that your PCP has provided?

☐ Yes

☐ No

1. Would you be willing to help us with our research involving a confidential group discussion about health concerns and how to access information?

☐ Yes

☐ No

If yes, please provide details so that we can contact you

Email____________________________________________________

Mobile_______________________
